# Association between serum urate level and carotid atherosclerosis: an insight from a post hoc analysis of the PRIZE randomised clinical trial

**DOI:** 10.1136/rmdopen-2022-002226

**Published:** 2022-04-10

**Authors:** Atsushi Tanaka, Shigeru Toyoda, Toru Kato, Hisako Yoshida, Shuichi Hamasaki, Masato Watarai, Tomoko Ishizu, Shinichiro Ueda, Teruo Inoue, Koichi Node, the PRIZE study investigators

**Affiliations:** 1 Department of Cardiovascular Medicine, Saga University, Saga, Japan; 2 Department of Cardiovascular Medicine, Dokkyo Medical University, Mibu, Japan; 3 Department of Cardiovascular Medicine, National Hospital Organisation Tochigi Medical Center, Utsunomiya, Japan; 4 Department of Medical Statistics, Osaka City University Graduate School of Medicine, Osaka, Japan; 5 Department of Cardiology, Imakiire General Hospital, Kagoshima, Japan; 6 Cardiovascular Center, Anjo Kosei Hospital, Anjo, Japan; 7 Department of Cardiology, Faculty of Medicine, University of Tsukuba, Tsukuba, Japan; 8 Department of Clinical Pharmacology and Therapeutics, University of the Ryukyus, Nishihara, Japan; 9 Center for Advanced Medical Science Research, Dokkyo Medical University, Mibu, Japan

**Keywords:** Gout, Cardiovascular Diseases, Atherosclerosis

## Abstract

**Objectives:**

Elevated serum urate (SU) levels are associated with arterial atherosclerosis and subsequent cardiovascular events. However, an optimal therapeutic target SU level for delaying atherosclerotic progression in patients with hyperuricaemia remains uncertain. The aim of this analysis was to assess an association between changes in SU level and carotid intima–media thickness (IMT) to examine whether an optimal SU concentration exists to delay atherosclerotic progression.

**Methods:**

This was a post hoc analysis of the PRIZE (programme of vascular evaluation under uric acid control by xanthine oxidase inhibitor, febuxostat: multicentre, randomised controlled) study of Japanese adults with asymptomatic hyperuricaemia. The primary endpoint of this analysis was an association between changes in SU levels and mean common carotid artery IMT (CCA-IMT) after 24 months of febuxostat treatment.

**Results:**

Among subjects treated with febuxostat (n=239), a total of 204 who had both data on SU and mean CCA-IMT at baseline and 24 months were included in this analysis. The mean baseline SU level was 7.7±1.0 mg/dL, and febuxostat treatment significantly reduced SU concentrations at 24 months (estimated mean change ‒3.051 mg/dL, 95% CI ‒3.221 to ‒2.882). A multivariable linear regression analysis revealed that a reduction in SU level was associated with changes in mean CCA-IMT values at 24 months (p=0.025). In contrast, the achieved SU concentrations were not associated with changes in mean CCA-IMT at 24 months.

**Conclusion:**

A greater reduction in SU, but not its achieved concentrations, may be associated with delayed progression of carotid IMT in patients with asymptomatic hyperuricaemia treated with febuxostat.

**Trial registration number:**

UMIN000012911

**Key messages**

**What is already known about this subject?**

- The therapeutic target serum urate (SU) level for delaying atherosclerotic progression in patients with hyperuricaemia has not been determined.

**What does this study add?**

- Greater SU reductions were associated with delayed carotid intima–media thickness (IMT) progression over 24 months of febuxostat treatment in Japanese patients with asymptomatic hyperuricaemia.
- The SU concentrations achieved with febuxostat treatment were not associated with changes in carotid IMT.
- Febuxostat treatment did not reduce high-sensitivity C reactive protein concentrations, irrespective of dose of febuxostat.

**How might this impact on clinical practice or further developments?**

- A greater reduction in SU level, but not the achieved concentration, is associated with delayed carotid IMT progression in patients with asymptomatic hyperuricaemia treated with febuxostat over 24 months.

## Introduction

Serum urate (SU) levels are positively associated with cardiometabolic disorders, such as hypertension and diabetes, and are involved in the pathophysiological development of cardiovascular disease (CVD).^1^ Accumulated evidence also suggests that an elevated SU level is an independent predictor of adverse cardiovascular outcomes in a broad range of individuals.^2–7^ The SU concentration is therefore considered a potential marker for CVD risk and a residual risk factor, although its causal relationship is complex. However, results of several observational and interventional studies have suggested that a beneficial effect of SU-lowering therapy on cardiovascular outcomes remains inconclusive.^8–14^ Moreover, results of previous cohort studies have demonstrated a J-shaped or U-shaped relationship between SU levels and mortality.^15–19^ Thus, cardiovascular benefits obtained from SU-lowering therapy remain controversial, and accordingly, an optimal therapeutic target SU concentration to improve cardiovascular outcomes and mortality remains to be established.

High SU levels promote arterial atherosclerotic changes and are associated with atherosclerosis in multiple vessels, such as the carotid, coronary and peripheral arteries.^20–24^ In some animal models, SU-lowering therapy attenuated experimental atherosclerosis via alleviation of inflammatory responses and excess reactive oxygen species (ROS).^25 26^ However, available evidence on the clinical effect of SU-lowering treatment on the burden of atherosclerosis in patients with hyperuricaemia is currently limited, and therapeutic target SU level for delaying atherosclerotic progression in that patient population is also uncertain.

The PRIZE (programme of vascular evaluation under uric acid control by xanthine oxidase (XO) inhibitor, febuxostat: multicentre, randomised controlled) study investigated whether SU-lowering therapy with febuxostat, a non-purine selective inhibitor of XO, can slow the progression of carotid atherosclerosis assessed as intima–media thickness (IMT) in patients with asymptomatic hyperuricaemia.^27^ In that study, 24 months of febuxostat treatment, compared with non-pharmacological treatments for hyperuricaemia, did not affect changes in carotid IMT. In this secondary analysis from the PRIZE study, we sought to assess an association between changes in the SU level and carotid atherosclerosis and examine whether there is an optimal therapeutic target SU concentration to delay progression.

## Methods

### Study design

Details of the PRIZE study design have been reported elsewhere.^27 28^ In brief, the study was an investigator-initiated multicentre, prospective, randomised, open-label, blinded-endpoint clinical trial. Recruitment and follow-up were conducted between May 2014 and August 2018 at 48 clinical sites in Japan (University Hospital Medical Information Network Clinical Trial Registry UMIN000012911).

Prior to enrolment, all participants received an adequate explanation of the study plan and provided written informed consent. Eligible participants were randomly assigned to either an add-on febuxostat group or a control group (non-pharmacological treatment of hyperuricaemia) in a 1:1 ratio at the web-based PRIZE Data Centre. Randomisation was carried out with a modified minimisation method with a biased-coin assignment balanced for age (<65, ≥65 years), gender, type 2 diabetes (yes, no), SU level (<8.0, ≥8.0 mg/dL) and maximum common carotid artery IMT (CCA-IMT: <1.3, ≥1.3 mm) in the screening period. Participants were then followed up over 24 months after the baseline visit.

This post hoc analysis was planned after publication of the main results of the PRIZE study,^27^ and the analysis plans of a series of PRIZE secondary analyses, including the present analysis, were subsequently registered (University Hospital Medical Information Network Clinical Trial Registry UMIN000041322).

### Study participants

The study inclusion and exclusion criteria are described elsewhere.^27 28^ Briefly, adults who had asymptomatic hyperuricaemia with an SU level >7.0 mg/dL and a maximum CCA-IMT ≥1.1 mm at the screening visit were included. Key exclusion criteria were the administration of any SU-lowering medications within 8 weeks before eligibility assessment, the presence of gouty tophus or gouty arthritis within 1 year prior to the assessment of eligibility, renal dysfunction (estimated glomerular filtration rate <30 mL/min 1.73 m^2^ or on dialysis), and the presence of recent-onset coronary artery disease or cerebrovascular disease within 3 months before enrollment.

All analyses were conducted in a modified intention-to-treat manner using the full analysis set (FAS), including all randomised subjects who had no serious violation of the study protocol.^27^ Among the FAS population treated with febuxostat (n=239), subjects who had both data on SU level and mean CCA-IMT at baseline and 24 months were included in the present analysis.

### Study treatment

Participants allocated to the febuxostat group received an initial dose of 10 mg daily that was planned to increase to 20 mg daily at 1 month and 40 mg daily (targeted maintenance dose) at 2 months. The dose could be increased to 60 mg daily at 3 months or later, according to individual levels of SU and local investigator’s judgement. Background therapies for comorbidities, such as hypertension, diabetes and dyslipidaemia, were planned to remain unchanged in principle during the study period.

### Outcome measures

The primary endpoint of the present analysis was the association between changes from baseline over 24 months of febuxostat treatment in SU levels and mean CCA-IMT. Secondary endpoints included the following: (1) association between changes from baseline to 24 months in SU levels and high-sensitivity C reactive protein (hs-CRP) and (2) relationship between the dose of febuxostat and IMT and hs-CRP at 24 months.

Data on carotid IMT and blood examinations collected at baseline, 12 months and 24 months after initiation of study intervention were used in the present analysis. Measurements of SU and hs-CRP were conducted at each local site and at central laboratories at SRL, Tokyo, Japan, respectively.

### Evaluation of carotid IMT

The detailed protocol and method for measuring carotid IMT was described previously.^27 28^ Based on the standardised protocol,^29^ high-resolution carotid ultrasonography using a standard system equipped with a >7.5 MHz linear transducer was performed at each study site in a blinded manner by a trained sonographer at baseline and 12 and 24 months. Longitudinal B-mode images, perpendicular to the ultrasound beam, with a 3–4 cm imaging depth, were recorded in the distal CCA at 10 mm from the carotid bulb.

The imaging data files were then collected and sent to a core imaging laboratory (Tsukuba Echo Core Laboratory), and the IMT values were measured independently by an expert analyser unaware of study allocation using an automated IMT measurement software program (Vascular Research Tools 5, Medical Imaging Applications, Coralville, Iowa, USA).^30^ The mean far-wall CCA-IMT on the left and right sides was averaged.

### Statistical analysis

For the baseline variables, summary statistics were expressed as number (%) for categorical data, mean and SD for normally distributed continuous variables and median and IQR for skewed variables. Mean values and 95% CIs of variables (SU concentration, mean CCA-IMT and hs-CRP) measured at baseline and 12 and 24 months and changes from baseline were predicted by the mixed-effect model with each value (SU concentration, mean CCA-IMT and hs-CRP) as an outcome variable, adjusting for age, sex and each corresponding value at baseline. To examine the relationship between changes in the SU concentrations and outcome measures (mean CCA-IMT and hs-CRP) at 24 months, we used a linear mixed-effect model adjusted for age, sex and each corresponding value at baseline. In addition, we examined whether those relationships differed depending on the median value of mean CCA-IMT at baseline and the final dose of febuxostat using a non-linear mixed-effect model with interaction.

All p values were two sided with a level of significance of 0.05, and there were no adjustments for multiple comparisons. All statistical analyses were performed using R V.4.0.1 (R Foundation for Statistical Computing, Vienna, Austria).

## Results

### Participant baseline demographics and clinical characteristics

**Figure 1:**
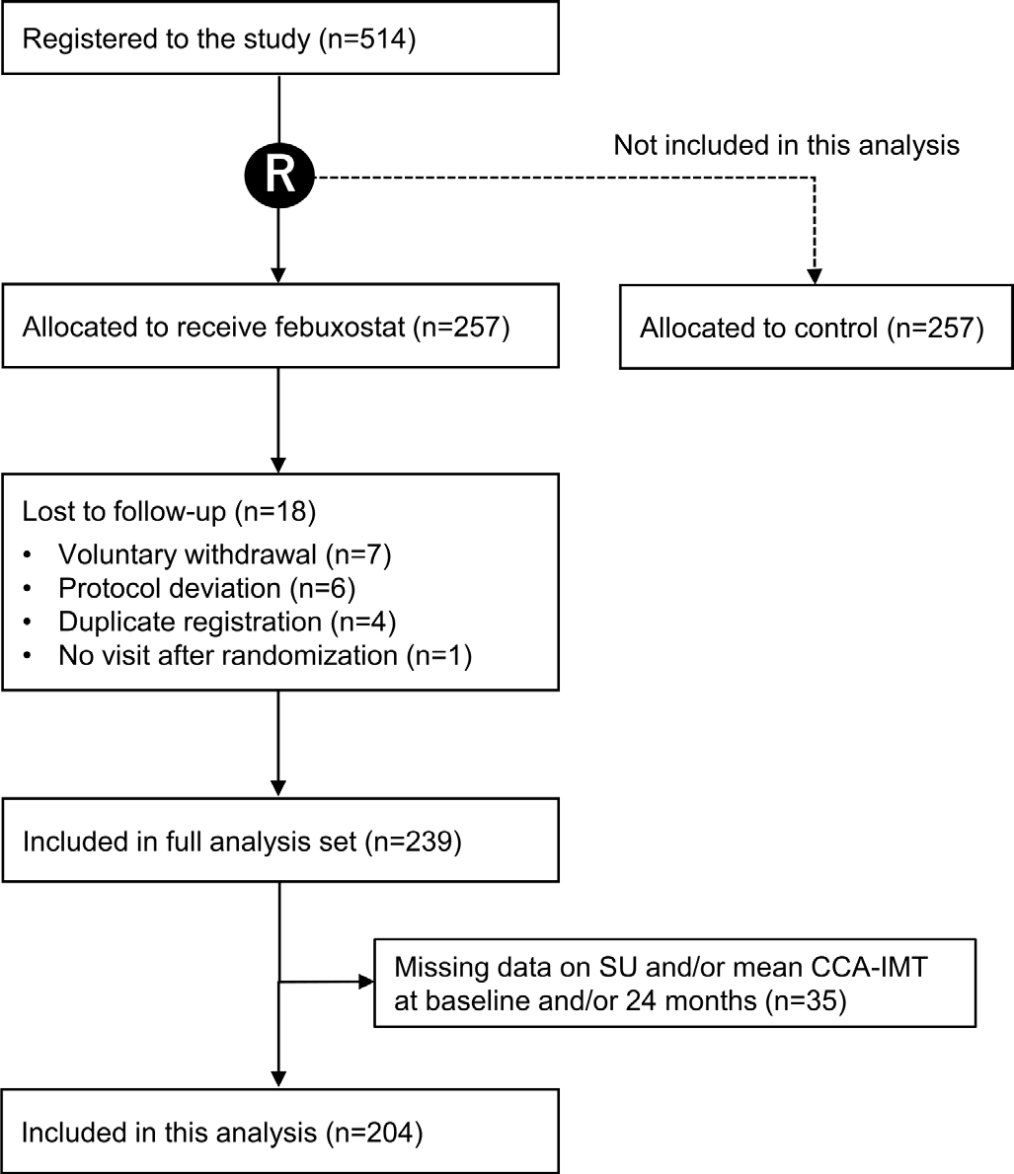
Flow chart of study subjects allocated to the febuxostat group and included in this analysis. CCA-IMT, common carotid artery intima–media thickness; SU, serum urate.

We included a total of 204 subjects treated with febuxostat in the present analysis (figure 1), and their demographic and clinical characteristics at baseline are shown in table 1. The mean age was 69.1±9.6 years, and one-fifth were female. Most had a history of treated hypertension and/or dyslipidaemia, while the proportion of subjects with a history of CVD was relatively small in the PRIZE study.

**Table 1:** Baseline demographics and clinical characteristics of subjects treated with febuxostat and included in this analysis

Variable	Treated with febuxostat (n=204)
Age, years	69.1±9.6
Female	41 (20.1)
Systolic blood pressure, mm Hg	128.8±14.9
Diastolic blood pressure, mm Hg	73.1±11.8
Body mass index, kg/m^2^	25.0±4.0
Previous history	
Hypertension	179 (87.7)
Diabetes mellitus	77 (37.7)
Dyslipidaemia	122 (59.8)
Gouty arthritis	5 (2.5)
Myocardial infarction	27 (13.2)
Stroke	12 (5.9)
Heart failure	36 (17.6)
Medication	
Renin–angiotensin system inhibitor	134 (65.7)
Calcium channel blocker	113 (55.4)
Beta-blocker	75 (36.8)
Diuretic	60 (29.4)
Statin	98 (48.0)
Antiplatelet agent	83 (40.7)

Data are expressed as mean±SD or number (percentage).

### Changes in SU concentration, CCA-IMT and hs-CRP

Among the subjects analysed, the mean SU concentration and mean CCA-IMT at baseline were 7.7±1.0 mg/dL and 0.830±0.176 mm, respectively, and the median hs-CRP level at baseline was 651 (IQR 355–1550) ng/mL. Estimated mean values and 95% CIs (SU concentration, mean CCA-IMT and hs-CRP) measured at each time point and changes from baseline are shown in table 2. The distribution of changes at 12 and 24 months are also shown in online supplemental figure S1. Febuxostat treatment significantly reduced SU concentrations at 12 months (estimated mean change ‒2.672 mg/dL, 95% CI ‒2.845 to ‒2.499) and 24 months (estimated mean change ‒3.051 mg/dL, 95% CI ‒3.221 to ‒2.882). Meanwhile, febuxostat treatment did not decrease mean CCA-IMT and hs-CRP concentrations over 24 months. The median dose of febuxostat at 24 months was 20^10 31^ mg daily; details are shown in online supplemental table S1. Among the 204 patients analysed, 80 (39.2%) were treated with ≥30 mg febuxostat daily.

10.1136/rmdopen-2022-002226.supp1 Supplementary data

**Table 2:** Change in SU concentration, mean CCA-IMT and hs-CRP level with febuxostat treatment over 24 months

Variable	Estimated mean value	95% CI
SU, mg/dL		
At 12 months	4.986	4.814 to 5.158
Change from baseline to 12 months	‒2.672	‒2.845 to ‒2.499
At 24 months	4.606	4.437 to 4.775
Change from baseline to 24 months	‒3.051	‒3.221 to ‒2.882
Mean CCA-IMT, mm		
At 12 months	0.818	0.804 to 0.831
Change from baseline to 12 months	0.004	‒0.009 to 0.017
At 24 months	0.820	0.807 to 0.833
Change from baseline to 24 months	0.006	‒0.006 to 0.019
Logarithmic hs-CRP		
At 12 months	6.681	6.533 to 6.829
Change from baseline to 12 months	0.203	0.054 to 0.351
At 24 months	6.651	6.506 to 6.797
Change from baseline to 24 months	0.173	0.027 to 0.318

CCA-IMT, common carotid artery intima–media thickness; hs-CRP, high-sensitivity C reactive protein; SU, serum urate.

### Association between SU levels and CCA-IMT values at 24 months

**Figure 2:**
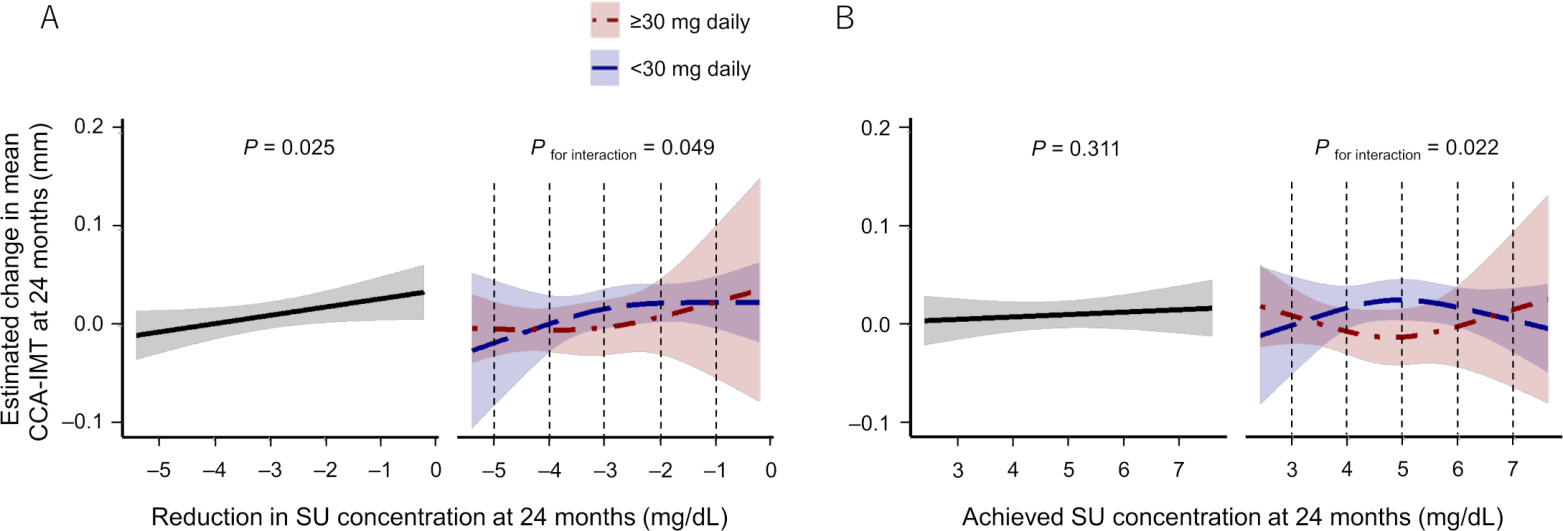
Association between SU levels and mean CCA-IMT values at 24 months. (A) Association between the reduction in SU concentration and the estimated changes in mean CCA-IMT values at 24 months. (B) Association between the achieved SU concentrations and the estimated changes in mean CCA-IMT values at 24 months. Left panels show the results in all overall subjects treated with febuxostat, and right panels show those in the subgroups stratified by the dose of febuxostat at 24 months (<30 mg daily: blue, ≥30 mg daily: red). Black dotted lines indicate corresponding SU levels that were analysed for differences in changes in mean CCA-IMT between subgroups stratified by final dose of febuxostat (see table 3). CCA-IMT, common carotid artery intima–media thickness; SU, serum urate.

A multivariable linear regression analysis revealed that a reduction in SU levels from baseline to 24 months was associated with changes in mean CCA-IMT values (p=0.025) (figure 2A, left panel). In contrast, there was no significant association between achieved SU concentrations and changes in mean CCA-IMT at 24 months (p=0.311) (figure 2B, left panel). These associations did not differ between the subgroups with high and low CCA-IMT at baseline (p for subgroup interaction >0.2, online supplemental figure S2). In addition, these associations remained even after adjustment for confounding cardiovascular risk factors; histories of hypertension, diabetes, dyslipidaemia, myocardial infarction and stroke (online supplemental figure S3) and even in another subgroup without previous gouty arthritis (n=199, online supplemental figure S4).

When stratified by the final dose of febuxostat at 24 months (<30 mg daily (low dose), ≥30 mg daily (high dose)), the global associations between SU levels and changes in the mean CCA-IMT differed between the two subgroups (p for subgroup interaction=0.049; figure 2A right panel, and p for subgroup interaction=0.022; figure 2B right panel). Meanwhile, changes in mean CCA-IMT examined at individual levels of reduction in SU and achieved SU concentration, except for 5.0 mg/dL, did not differ between subgroups (table 3).

**Table 3:** Differences in changes in outcome measures over 24 months according to levels of SU between subgroups stratified by final dose of febuxostat

Outcome measure	Reduction in SU concentration at 24 months (mg/dL)	Group difference*	95% CI	Achieved SU concentration at 24 months (mg/dL)	Group difference*	95% CI
Mean CCA-IMT, mm	‒5.0	0.015	‒0.055 to 0.084	3.0	0.009	‒0.046 to 0.064
	‒4.0	‒0.006	‒0.042 to 0.029	4.0	‒0.025	‒0.057 to 0.006
	‒3.0	‒0.019	‒0.054 to 0.015	5.0	‒0.038	‒0.074 to ‒0.003
	‒2.0	‒0.014	‒0.057 to 0.028	6.0	‒0.019	‒0.066 to 0.029
	‒1.0	0.000	‒0.082 to 0.083	7.0	0.012	‒0.076 to 0.100
Log-scaled hs-CRP	‒5.0	‒0.426	‒1.258 to 0.406	3.0	0.126	‒0.479 to 0.731
	‒4.0	‒0.043	‒0.465 to 0.380	4.0	0.082	‒0.280 to 0.444
	‒3.0	0.183	‒0.222 to 0.587	5.0	0.001	‒0.406 to 0.409
	‒2.0	0.047	‒0.458 to 0.552	6.0	‒0.131	‒0.654 to 0.392
	‒1.0	‒0.277	‒1.261 to 0.707	7.0	‒0.281	‒1.222 to 0.660

*Group differences were calculated as the subgroup treated with high-dose febuxostat (≥30 mg daily) minus that with low-dose febuxostat (<30 mg daily).

CCA-IMT, common carotid artery intima–media thickness; hs-CRP, high-sensitivity C reactive protein; SU, serum urate.

### Association between SU and hs-CRP concentrations at 24 months

**Figure 3:**
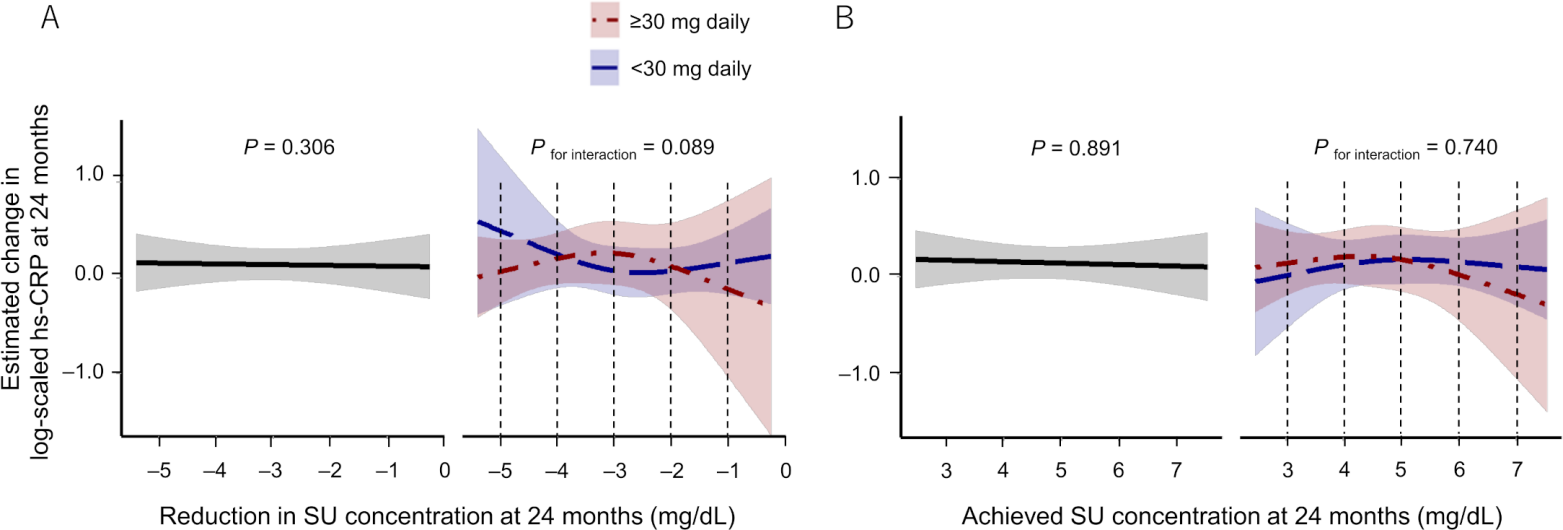
Association between SU levels and hs-CRP concentrations at 24 months. (A) Association between the reduction in SU concentration and the estimated changes in log-scaled hs-CRP concentrations at 24 months. (B) Association between the achieved SU concentrations and the estimated changes in log-scaled hs-CRP concentrations at 24 months. Left panels show the results in all overall subjects treated with febuxostat, and right panels show those in the subgroups stratified by the dose of febuxostat at 24 months (<30 mg daily: blue, ≥30 mg daily: red). Black dotted lines indicate corresponding SU levels that were analysed for differences in changes in log-scaled hs-CRP between subgroups stratified by final dose of febuxostat (see table 3). hs-CRP, high-sensitivity C reactive protein; SU, serum urate.

There was no significant association of SU-related parameters with change in log-scaled hs-CRP concentrations at 24 months (figure 3A, B left panels). Neither differed between subgroups (figure 3A, B right panels), and changes in log-scaled hs-CRP concentrations at individual levels of reduction in SU and achieved SU concentration also did not differ between subgroups (table 3).

## Discussion

Major findings of the present analysis are as follows: (1) greater SU reduction was associated with delayed carotid IMT progression over 24 months of febuxostat treatment in Japanese patients with asymptomatic hyperuricaemia, (2) the SU concentrations achieved with febuxostat treatment were not associated with changes in carotid IMT, (3) the baseline burdens of carotid IMT and cardiovascular risk factors did not affect those associations, (4) febuxostat treatment did not reduce hs-CRP concentrations despite significant decreases in SU levels and (5) high-dose febuxostat (≥30 mg daily) did not necessarily have a greater effect on the outcome measures than low-dose febuxostat (<30 mg daily). These findings suggest that a greater reduction in SU levels is helpful to delay the progression of carotid IMT in patients with hyperuricaemia, although the underlying mechanism remains unclear. Meanwhile, an optimal therapeutic target SU concentration for delaying carotid IMT progression was not identified in this analysis.

Accumulated evidence shows that hyperuricaemia is closely associated with cardiometabolic disorders, including hypertension and diabetes, and an increased risk of CVD and mortality.^1–7 32^ Thus, hyperuricaemia is a residual risk factor and a potential marker of adverse cardiovascular events. In several guidelines for the management of gout in the USA and Europe, initiation of SU-lowering medication is recommended for patients with symptomatic hyperuricaemia.^33–35^ A target level of SU (eg,<6.0 mg/dL and <5.0 mg/dL in patients with severe gout) is also recommended from the point of crystal dissolution.^33 36^ In contrast, in the Japanese guideline for the management of hyperuricaemia and gout,^37^ that therapy can be initiated even in patients with asymptomatic hyperuricaemia. Thus, although some vary by region, relevant guidelines provide an approach for initiating and managing SU-lowering therapy. However, no target SU concentration is currently established, at least in patients with asymptomatic hyperuricaemia. Furthermore, no optimal approach and target SU concentration have been determined for reducing atherosclerosis and the risk of subsequent CVD events in patients with hyperuricaemia regardless of gout status.

According to the results obtained from recent randomised clinical trials and meta analyses, whether SU-lowering medication is effective for preventing the development of CVD and mortality in patients with hyperuricaemia remains controversial.^12–14^ In addition, results of several cohort studies demonstrated a J-shaped or U-shaped relationship between SU levels and mortality.^15–19^ The most recent analysis from a randomised clinical trial (Febuxostat for Cerebral and CaRdiorenovascular Events PrEvEntion StuDy) also demonstrated a J-shaped relationship between SU levels and the incidence of composite cardiorenovascular events and mortality, with a nadir of estimated risk at achieved SU concentrations of >5.0 to ≤6.0 mg/dL, in elderly patients with asymptomatic hyperuricaemia at risk for cardiorenovascular events.^38^ These findings may, at least in part, support the aforementioned target SU concentration recommended in the relevant guidelines^33^ and suggest the possibility that excessive lowering of SU has a negative impact on cardiovascular and even non-cardiovascular systems. However, whether those estimated relationships between SU levels and incident hard end points can be applied to the relationship between SU levels and local atherosclerosis is poorly understood.

We previously examined the cross-sectional and longitudinal associations between SU levels and pathophysiological vascular properties indicative of atherosclerotic progression, such as endothelial function, arterial stiffness and carotid atherosclerosis, using data obtained from a multicentre, prospective, observational study of general subjects with treated hypertension.^39^ In the cross-sectional analysis, a lower level of SU was associated with better vascular function and lower CCA-IMT, partly dependent on gender. In the longitudinal analysis, the SU level at baseline was inversely associated with endothelial function assessed as flow-mediated vasodilatation both at 1.5 and 3.0 years after study recruitment. Accordingly, we speculated that lower levels of SU were harmless for vascular health and atherosclerotic progression, at least in that study population. Importantly, that study had an observational design and did not intend to include specifically patients with hyperuricaemia. However, the effects of SU-lowering therapy on vascular function and atherosclerosis in patients with hyperuricaemia remain controversial, and clinical evidence to determine a therapeutic target SU concentration from such a vascular perspective is also lacking.

Controversy currently exists regarding the value of carotid IMT as a clinical indicator of atherosclerosis and its impact on cardiovascular risk estimation.^31 40^ The extent of intervention effects on carotid IMT progression has been associated with the degree of reduction in the risk of cardiovascular events.^41^ The strength of the present analysis is that it is the first to investigate the association between changes in SU levels and carotid IMT specifically in hyperuricaemic patients treated with febuxostat. Twenty-four months after initiation of febuxostat treatment, a greater reduction in SU concentration was associated with slower progression of mean CCA-IMT, without a J-shaped or U-shaped relationship. Meanwhile, no target SU concentration for attenuation of carotid atherosclerosis was determined in this analysis. Interestingly, in a previously reported subgroup analysis stratified by baseline clinical characteristics from the original PRIZE study,^27^ febuxostat treatment tended to decrease the mean CCA-IMT in a subgroup of patients with a high SU level (≥8.0 mg/dL) at baseline relative to a subgroup with a low SU level (<8.0 mg/dL) at baseline, although the p value for subgroup interaction (0.094) was not statistically significant. Taken together, our findings suggest that greater SU-lowering may be favourable for delaying carotid IMT progression, especially in patients with a highly elevated level of SU (eg, ≥8.0 mg/dL), possibly contributing to the reduction in cardiovascular risk.

In the PRIZE study, febuxostat treatment, compared with non-pharmacological treatments for hyperuricaemia, did not delay carotid atherosclerosis progression.^27^ As the cardiovascular effects of hyperuricaemia seem to progress slowly,^1^ the 24-month study period might have been too short to determine the impact and treatment effects of febuxostat on carotid IMT. Nevertheless, according to the results of the present analysis, carotid atherosclerosis attenuation in patients with hyperuricaemia may depend on the degree of reduction in the SU concentration, even in this limited time period. Given the J-shaped or U-shaped relationship between SU levels and hard endpoints, further study is needed to determine the optimal therapeutic target SU level to reduce the overall risk of atherosclerosis, CVD events and mortality.

In the process of urate production, ROSs are simultaneously produced by XO catalysis. Intracellular urate also generates ROS and activates inflammasomes and several proinflammatory signalling pathways, resulting in proatherogenic responses.^42^ Therefore, it would be theoretically reasonable that chronic inflammation and atherosclerosis progression are attenuated by SU-lowering via pharmacological XO inhibition. However, the levels of hs-CRP, a representative marker indicative of inflammatory status, were unchanged by febuxostat treatment, even at high doses, despite a substantial decrease in the SU concentration. The precise reasons for this observation, which conflicts with the decrease in the inflammatory responses seen in animal models,^25 26 43^ are not known. In non-primate animals, the presence of active uricase results in significantly lower SU levels and greater sensitivity to the induction of hyperuricaemia. Non-primate animals may be, therefore, more sensitive to the inflammatory response to the induced hyperuricaemia. In addition, the levels of hs-CRP are not only elevated by urate-stimulated inflammation, but are also affected by age, gender and various health status-related factors, such as comorbidities.^44 45^ Thus, surrogate biomarkers would need to be determined more specifically to reflect urate activity and changes in urate activity with SU-lowering therapies.

### Limitations

The present analysis has several limitations. First, this was a post hoc analysis of the PRIZE study of Japanese patients with asymptomatic hyperuricaemia, and the sample size was not estimated to assess the aim of this sub analysis. In addition, the reproducibility of the results is uncertain in patients of other ethnicities, in different study designs, and with other SU-lowering therapies. Further, the baseline levels of SU were relatively low (mean 7.7±1.0 mg/dL). Additionally, changes in mean CCA-IMT over 24 months were very small (estimated mean 0.006 mm). Therefore, the ranges to assess the relationship between those changes were restricted to some extent. Finally, the final doses of febuxostat (median 20 mg daily) were also lower than expected. Despite the study protocol of febuxostat uptitration,^27 28^ dosage adjustments were left to the judgement of the local investigators. Moreover, the final doses of febuxostat, including 0 mg daily, do not account for medication compliance and the dose adjustment process during the study interval.

### Conclusion

Our findings suggest that a greater reduction in SU level, but not the achieved concentrations, is associated with delaying carotid IMT progression in patients with asymptomatic hyperuricaemia treated with febuxostat. Further study is needed to determine the optimal therapeutic target SU levels to reduce the overall risk of atherosclerosis, CVD events and mortality.

## Data Availability

Data are available on reasonable request. The data that support the findings of this study are available from the corresponding author on reasonable request.
