# Supplementary material for: Association between serum urate level and carotid atherosclerosis: an insight from a post hoc analysis of the PRIZE randomised clinical trial
Source: RMD Open. 2022 Apr 10;8(1):e002226. doi: 10.1136/rmdopen-2022-002226 (PMC9003608; doi:10.1136/rmdopen-2022-002226)
Supplement: Supplementary data [file rmdopen-2022-002226supp001.pdf]

Supplementary material online

Association between serum urate level and carotid atherosclerosis: an insight from a *post-hoc* analysis of the PRIZE randomized clinical trial

Atsushi Tanaka[1], Shigeru Toyoda[2], Toru Kato[3], Hisako Yoshida[4], Shuichi Hamasaki[5], Masato Watarai[6], Tomoko Ishizu[7], Shinichiro Ueda[8], Teruo Inoue[9], Koichi Node[1]; for the PRIZE study investigators

[1] Department of Cardiovascular Medicine, Saga University, Saga, Japan.
[2] Department of Cardiovascular Medicine, Dokkyo Medical University, Mibu, Japan.
[3] Department of Clinical Research, National Hospital Organization, Tochigi Medical Center, Utsunomiya, Japan.
[4] Department of Medical Statistics, Osaka City University Graduate School of Medicine, Osaka, Japan.
[5] Department of Cardiology, Imakiire General Hospital, Kagoshima, Japan.
[6] Cardiovascular Center, Anjo Kosei Hospital, Anjo, Japan.
[7] Department of Cardiology, Faculty of Medicine, University of Tsukuba, Tsukuba, Japan.
[8] Department of Clinical Pharmacology and Therapeutics, University of the Ryukyus, Nishihara, Japan.
[9] Center for Advanced Medical Science Research, Dokkyo Medical University, Mibu, Japan.

Text S1. The PRIZE Study Organization and Investigators

Principal Investigator: Koichi Node, MD, PhD (Saga University, Saga, Japan).
PRIZE Steering Committee: Toyoaki Murohara, MD, PhD (Nagoya University Graduate School of Medicine, Nagoya, Japan), Teruo Inoue, MD, PhD (Dokkyo Medical University, Mibu, Japan), Masataka Sata, MD, PhD (Tokushima University Graduate School, Tokushima, Japan), Mitsuru Ohishi, MD, PhD (Kagoshima University, Kagoshima, Japan).
PRIZE Executive Committee: Kotaro Yokote, MD, PhD (Chiba University Graduate School of Medicine, Chiba, Japan), Kazuomi Kario, MD, PhD (Jichi Medical University School of Medicine, Shimotsuke, Japan), Hirotaka Watada, MD, PhD (Juntendo University Graduate School of Medicine, Tokyo, Japan), Iichiro Shimomura, MD, PhD (Osaka University, Graduate School of Medicine, Suita, Japan), Munehide Matsuhisa, MD, PhD (Tokushima University Graduate School, Tokushima, Japan), Yoshihiro Fukumoto, MD, PhD (Kurume University School of Medicine, Kurume, Japan), Koji Maemura, MD, PhD (Nagasaki University Graduate School of Biomedical Sciences, Nagasaki, Japan), Yusuke Ohya, MD, PhD (University of the Ryukyus, Okinawa, Japan).
PRIZE Site Investigators: Yuichi Akasaki (Kagoshima University, Kagoshima, Japan), Junya Ako (Kitasato University School of Medicine, Sagamihara, Japan), Hirohisa Amano (Dokkyo Medical University, Mibu, Japan), Kazutaka Aonuma (Graduate School of Comprehensive Human Sciences, University of Tsukuba, Tsukuba, Japan), Yutaka Aoyama (Nagoya Daini Red Cross Hospital, Nagoya, Japan), Hirofumi Arai (Kameda Medical Center, Komogawa, Japan), Kuniya Asai (Nippon Medical School, Tokyo, Japan), Machiko Asaka (Saga University, Saga, Japan), Yoshifumi Awaji (Nagoya Ekisaikai Hospital, Nagoya, Japan), Noriko Ban (Chiba Aoba Municipal Hospital, Chiba, Japan), Toshiaki Ban (Isumi Medical Center, Isumi, Japan), Yasuko K Bando (Nagoya University Graduate School of Medicine, Nagoya, Japan), Hiroyuki Daida (Juntendo University Graduate School of Medicine, Tokyo, Japan), Shunsuke Eguchi (Japanese Red Cross Nagoya Daini Hospital, Nagoya, Japan), Mami Enomoto (Graduate School of Comprehensive Human Sciences, University of Tsukuba, Tsukuba, Japan), Yuichi Fujii (Hiroshima General Hospital of West Japan Railway Company, Hiroshima, Japan), Akinori Fujikake (Dokkyo Medical University Saitama Medical Center, Koshigaya, Japan), Masanori Fujimoto (Graduate School of Medicine, Chiba University, Chiba, Japan), Tomohiro Fujisaka (Osaka Medical College, Takatsuki, Japan), Shuichi Fujita (Osaka Medical College, Takatsuki, Japan), Satoki Fukae (Nagasaki University

Graduate School of Biomedical Sciences, Nagasaki, Japan), Daiju Fukuda (Tokushima University Graduate School of Biomedical Sciences, Tokushima, Japan), Mieko Fukui (Kimitsu Chuo Hospital, Kisarazu, Japan), Yuhei Goriki (Miyazaki Medical Association Hospital, Miyazaki, Japan), Shuichi Hamasaki (Kagoshima City Hospital, Kagoshima, Japan), Tomoya Hara (Tokushima University Graduate School of Biomedical Sciences, Tokushima, Japan), Hiroshi Hasegawa (Chiba University Graduate School of Medicine, Chiba, Japan), Kenichi Hashimoto (National Defense Medical College, Tokorozawa, Japan), Mitsumasa Hata (Sekino Hospital, Tokyo, Japan), Shiro Hata (Sasebo City General Hospital, Sasebo, Japan), Ryo Hayashida (Nagoya University Graduate School of Medicine, Nagoya, Japan), Akihiro Higashi (Dokkyo Medical University Saitama Medical Center, Koshigaya, Japan), Seiichiro Higuchi (Graduate School of Medicine, Chiba University, Chiba, Japan), Akihiro Honda (Kurume University School of Medicine, Kurume, Japan), Satoshi Hoshide (Jichi Medical University School of Medicine, Shimotsuke, Japan), Masaaki Hoshiga (Osaka Medical College, Takatsuki, Japan), Junko Hotchi (Tokushima University Graduate School of Biomedical Sciences, Tokushima, Japan), Sachiyo Igata (Kurume University School of Medicine, Kurume, Japan), Yumi Ikehara (University of the Ryukyus, Nishihara, Japan), Teruo Inoue (Dokkyo Medical University, Mibu, Japan), Youhei Inoue (Miyazaki Medical Association Hospital, Miyazaki, Japan), Hiroko Ishigami (Nagoya Daini Red Cross Hospital, Nagoya, Japan), Masaharu Ishihara (Hyogo College of Medicine, Nishinomiya, Japan), Hideki Ishii (Nagoya University Graduate School of Medicine, Nagoya, Japan), Tetsuya Ishikawa (Dokkyo Medical University Saitama Medical Center, Koshigaya, Japan), Takashi Ishimatsu (Nagasaki University Graduate School of Biomedical Sciences, Nagasaki, Japan), Yusuke Ishiyama (Jichi Medical University School of Medicine, Shimotsuke, Japan), Takahide Ito (Osaka Medical College, Takatsuki, Japan), Ayumi Ito (Nagoya Daini Red Cross Hospital, Nagoya, Japan), Toshiaki Kadokami (Fukuoka Saiseikai Futsukaichi Hospital, Chikushino, Japan), Haruo Kamiya (Japanese Red Cross Nagoya Daiichi Hospital, Nagoya, Japan), Soichiro Kashihara (Fukuoka Saiseikai Futsukaichi Hospital, Chikushino, Japan), Yoshihiro Kawamura (Kasugai Municipal Hospital, Kasugai, Japan), Kazuo Kitagawa (Tokyo Women's Medical University, Tokyo, Japan), Yoshio Kobayashi (Chiba University Graduate School of Medicine, Chiba, Japan), Satoshi Kodera (Asahi General Hospital, Asahi, Japan), Seiji Koga (Nagasaki University Graduate School of Biomedical Sciences, Nagoya, Japan), Hisashi Koide (Chiba University Graduate School of Medicine, Chiba, Japan), Yuji Koide (Nagasaki University Graduate School of Biomedical Sciences, Nagasaki, Japan), Hiroshi Koiwaya (Miyazaki Medical Association Hospital, Miyazaki, Japan),

Hiroki Kojima (Nagoya University Graduate School of Medicine, Nagoya, Japan), Eri Komai (Graduate School of Medicine, Chiba University, Chiba, Japan), Takaaki Komatsu (Dokkyo Medical University Saitama Medical Center, Koshigaya, Japan), Shingo Kono (Kobe City Medical Center General Hospital, Kobe, Japan), Takashi Kono (Graduate School of Medicine, Chiba University, Chiba, Japan), Yoshiaki Kubota (Nippon Medical School, Tokyo, Japan), Akio Kuroda (Institute of Advanced Medical Sciences, Tokushima University, Tokushima, Japan), Takanori Kuroyanagi (Dokkyo Medical University Saitama Medical Center, Koshigaya, Japan), Akifumi Kushiyama (The Institute for Adult Diseases, Asahi Life Foundation, Tokyo, Japan), Kenya Kusunose (Tokushima University Graduate School of Biomedical Sciences, Tokushima, Japan), Tatsuya Maruhashi (Graduate School of Biomedical and Health Sciences, Hiroshima University, Hiroshima, Japan), Kazuo Matsunaga (Imari Arita Kyoritsu Hospital, Matsuura, Japan), Tomomi Matsuura (Tokushima University Graduate School of Biomedical Sciences, Tokushima, Japan), Takafumi Mayama (Graduate School of Medicine, Chiba University, Chiba, Japan), Daigo Mine (Saga-Ken Medical Centre Koseikan, Saga, Japan), Masatoshi Miyamura (Osaka Medical College, Takatsuki, Japan), Ryota Morimoto (Nagoya University Graduate School of Medicine, Nagoya, Japan), Hideaki Morita (Osaka Medical College, Takatsuki, Japan), Hidekazu Nagano (Chiba University Graduate School of Medicine, Chiba, Japan), Hidemitsu Nakagawa (Nozaki Tokushukai Hospital, Daito, Japan), Katsunori Nakamura (Ryukyu University Hospital, Nishihara, Japan), Ryo Nakamura (Fukuoka Saiseikai Futsukaichi Hospital, Chikushino, Japan), Ikuko Nakamura (Saga-Ken Medical Centre Koseikan, Saga, Japan), Hitoshi Nakashima (National Hospital Organization Kagoshima Medical Center, Kagoshima, Japan), Mamoru Nanasato (Japanese Red Cross Nagoya Daini Hospital, Nagoya, Japan), Isao Nishi (National Hospital Organization Kasumigaura Medical Center, Tsuchiura, Japan), Shinichi Niwano (Kitasato University School of Medicine, Sagamihara, Japan), Shuichi Nomura (Hiroshima General Hospital of West Japan Railway Company, Hiroshima, Japan), Nozomu Oda (Graduate School of Biomedical and Health Sciences, Hiroshima University, Hiroshima, Japan), Shio Oguchi (Kasugai Municipal Hospital, Kasugai, Japan), Mitsutoshi Oguri (Kasugai Municipal Hospital, Kasugai, Japan), Arihide Okahara (Saga-Ken Medical Centre Koseikan, Saga, Japan), Masaaki Okutsu (Nozaki Tokushukai Hospital, Daito, Japan), Fumitake Ozaki (Dokkyo Medical University Saitama Medical Center, Koshigaya, Japan), Michishige Ozeki (Osaka Medical College, Takatsuki, Japan), Tomoko Saisu (Tokyo Medical University, Tokyo, Japan), Yuichi Saito (Chiba University Hospital, Chiba, Japan), Makoto Saitoh (Nishio Municipal Hospital, Nishio, Japan), Yosuke Saka (Kasugai Municipal Hospital,

Kasugai, Japan), Yoshihiko Sakai (Dokkyo Medical University Saitama Medical Center, Koshigaya, Japan), Kazushi Sakane (Osaka Medical College, Takatsuki, Japan), Ikki Sakuma (Graduate School of Medicine, Chiba University, Chiba, Japan), Shakya Sandeep (Asahi General Hospital, Asahi, Japan), Hiroaki Sano (Nagoya Ekisaikai Hospital, Nagoya, Japan), Hisakuni Sekino (Sekino Hospital, Tokyo, Japan), Yuka Senoo (Nagoya Daini Red Cross Hospital, Nagoya, Japan), Kensaku Shibata (Osaka Medical College, Takatsuki, Japan), Yoshisato Shibata (Miyazaki Medical Association Hospital, Miyazaki, Japan), Takahisa Shibata (Isumi Medical Center, Isumi, Japan), Akina Shiga (Graduate School of Medicine, Chiba University, Chiba, Japan), Kazuki Shiina (Tokyo Medical University, Tokyo, Japan), Michio Shimabukuro (Tokushima University Graduate School of Biomedical Sciences, Tokushima, Japan), Yusaku Shimbo (Nagoya University Graduate School of Medicine, Nagoya, Japan), Wataru Shimizu (Nippon Medical School, Tokyo, Japan), Masahisa Shimpo (Jichi Medical University School of Medicine, Shimotsuke, Japan), Takeshi Soeki (Tokushima University Graduate School of Biomedical Sciences, Tokushima, Japan), Koichi Sohmiya (Osaka Medical College, Takatsuki, Japan), Hiroyuki Suzuki (Nagoya Daini Red Cross Hospital, Nagoya, Japan), Susumu Suzuki (Nagoya University Graduate School of Medicine, Nagoya, Japan), Makoto Suzuki (Kameda Medical Center, Kamogawa, Japan), Nobuhiro Tahara (Kurume University School of Medicine, Kurume, Japan), Tazu Tahara (The Institute for Adult Diseases, Asahi Life Foundation, Tokyo, Japan), Sadako Takahashi (Jichi Medical University School of Medicine, Shimotsuke, Japan), Bonpei Takase (National Defense Medical College, Tokorozawa, Japan), Kaoru Takegami (Saga-Ken Medical Centre Koseikan, Saga, Japan), Tomoko Takiguchi (Kimitsu Chuo Hospital, Kisarazu, Japan), Tomonobu Takikawa (Kasugai Municipal Hospital, Kasugai, Japan), Ai Tamura (Graduate School of Medicine, Chiba University, Chiba, Japan), Tomoaki Tanaka (Chiba University Graduate School of Medicine, Chiba, Japan), Akihito Tanaka (Nagoya University Graduate School of Medicine, Nagoya, Japan), Hiroyuki Tanaka (Niko Clinic, Takeo, Japan), Jun Tanigawa (Osaka Medical College, Takatsuki, Japan), Daisuke Tanimura (Nagoya Ekisaikai Hospital, Nagoya, Japan), Yosuke Tatami (Nagoya University Graduate School of Medicine, Nagoya, Japan), Takashi Terano (Chiba Aoba Municipal Hospital, Chiba, Japan), Fumio Terasaki (Osaka Medical College, Takatsuki, Japan), Tomoyuki Tobushi (Fukuoka Saiseikai Futsukaichi Hospital, Chikushino, Japan), Seiko Tokoi (Dokkyo Medical University, Mibu, Japan), Toshiyuki Tsubouchi (Nozaki Tokushukai Hospital, Daito, Japan), Daigaku Uchida (Hotaruno Central Clinic, Kisarazu, Japan), Tomohiro Ueda (Hiroshima General Hospital of West Japan Railway Company, Hiroshima, Japan), Rie Ueno

(Tokushima University Graduate School of Biomedical Sciences, Tokushima, Japan), Hiromi Ueno (Jichi Medical University School of Medicine, Shimotsuke, Japan), Chikara Ueyama (Gifu Prefectural Tajimi Hospital, Tajimi, Japan), Tetsuzo Wakatsuki (Tokushima University Graduate School of Biomedical Sciences, Tokushima, Japan), Tomohiko Watanabe (Osaka Medical College, Takatsuki, Japan), Masato Watarai (Anjo Kosei Hospital, Anjo, Japan), Isao Yaguchi (Dokkyo Medical University Saitama Medical Center, Koshigaya, Japan), Ayumu Yajima (Saga University, Saga, Japan), Jiko Yamada (Tokyo Medical University, Tokyo, Japan), Kyohei Yamamoto (Chiba Aoba Municipal Hospital, Chiba, Japan), Sachiko Yamauchi (Ryukyu University Hospital, Nishihara, Japan), Yohei Yamauchi (Osaka Medical College, Takatsuki, Japan), Naoto Yokota (Yokota Naika, Miyazaki, Japan), Tomohiko Yoshida (Chiba Aoba Municipal Hospital, Chiba, Japan), Goro Yoshioka (Miyazaki Medical Association Hospital, Miyazaki, Japan).

Members of the Data and Safety Monitoring Board: Hiroyuki Daida, MD, PhD (Juntendo University Graduate School of Medicine, Tokyo, Japan); Junya Ako, MD, PhD, (Kitasato University School of Medicine, Sagamihara, Japan); Kazuo Kitagawa, MD, PhD (Tokyo Women's Medical University, Tokyo, Japan).

Members of the Clinical Events Committee: Wataru Shimizu, MD, PhD (Nippon Medical School, Tokyo, Japan); Yoshio Kobayashi, MD, PhD (Chiba University Graduate School of Medicine, Chiba, Japan); Masaharu Ishihara, MD, PhD (Hyogo College of Medicine, Nishinomiya, Japan).

Imaging Core Laboratory: Tsukuba Echo Core Laboratory. LLC; Tomoko Ishizu, MD, PhD (Tsukuba University, Tsukuba, Japan).

Statistics: Shusuke Tani, PhD (Nouvelle Place Inc., Tokyo, Japan); Hisako Yoshida, PhD (Organization for Clinical Medicine Promotion, Tokyo, Japan).

Monitoring: Shinichiro Ueda, MD, PhD (Clinical Research Management Center, University of the Ryukyus, Okinawa, Japan)

Audit Team: Clinical Research Support Center, University of the Ryukyus, Okinawa, Japan.

Study Secretariat: Atsushi Tanaka, MD, PhD (Saga University, Saga, Japan), Jun-ichi Oyama, MD, PhD (Saga University, Saga, Japan), Ms. Mikiko Kagiyama (Saga University, Saga, Japan); Ms. Itsuka Suzuki (Nouvelle Place Inc., Tokyo, Japan); Ms. Keiko Onodera (Organization for Clinical Medicine Promotion, Tokyo, Japan).

Figure S1. Distributions of changes in SU concentrations, mean CCA-IMT, and log-scaled hs-CRP at 12 and 24 months

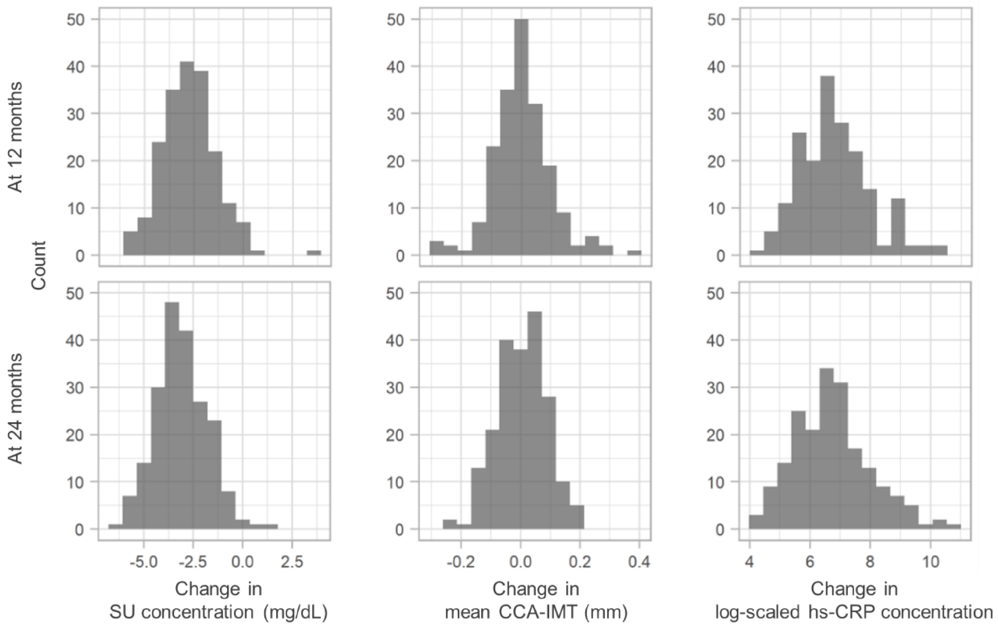

CCA-IMT, common carotid artery intima-media thickness; hs-CRP, high-sensitivity C-reactive protein; SU, serum urate.

Figure S2. Association between SU levels and mean CCA-IMT values at 24 months stratified by median value of baseline mean CCA-IMT

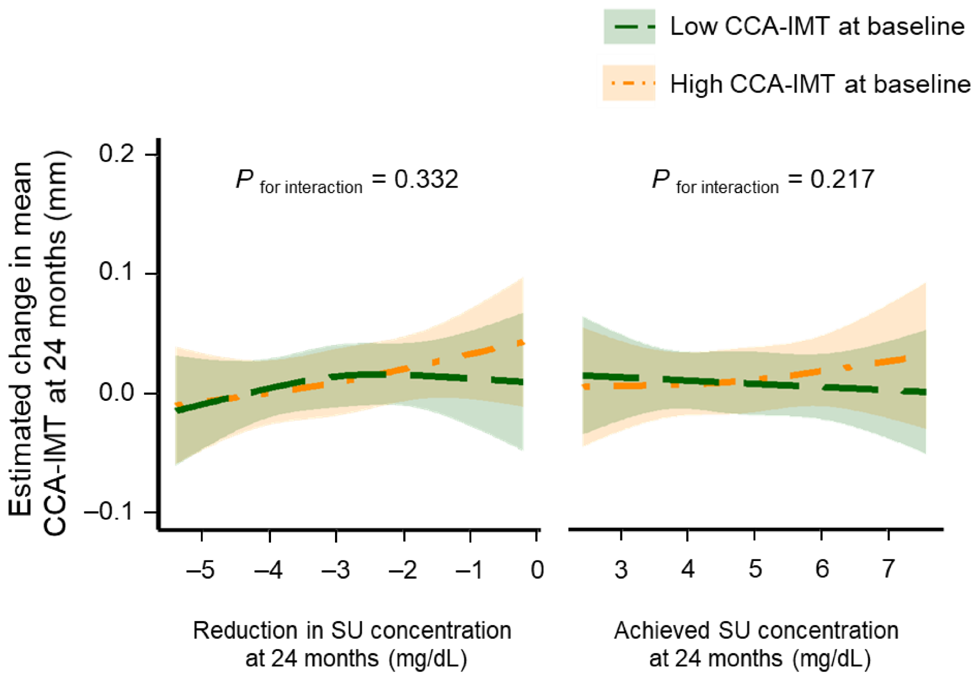

Association between SU levels (right, reduction in SU concentration; left, achieved SU concentrations) and mean CCA-IMT values at 24 months stratified by the median value (0.7975 mm) of mean CCA-IMT at baseline (low: green, high: orange).
CCA-IMT, common carotid artery intima-media thickness; SU, serum urate.

Figure S3. Association between SU levels and mean CCA-IMT values at 24 months adjusted by age, sex, baseline mean CCA-IMT, and history of hypertension, diabetes, dyslipidemia, and CVD (myocardial infarction and stroke)

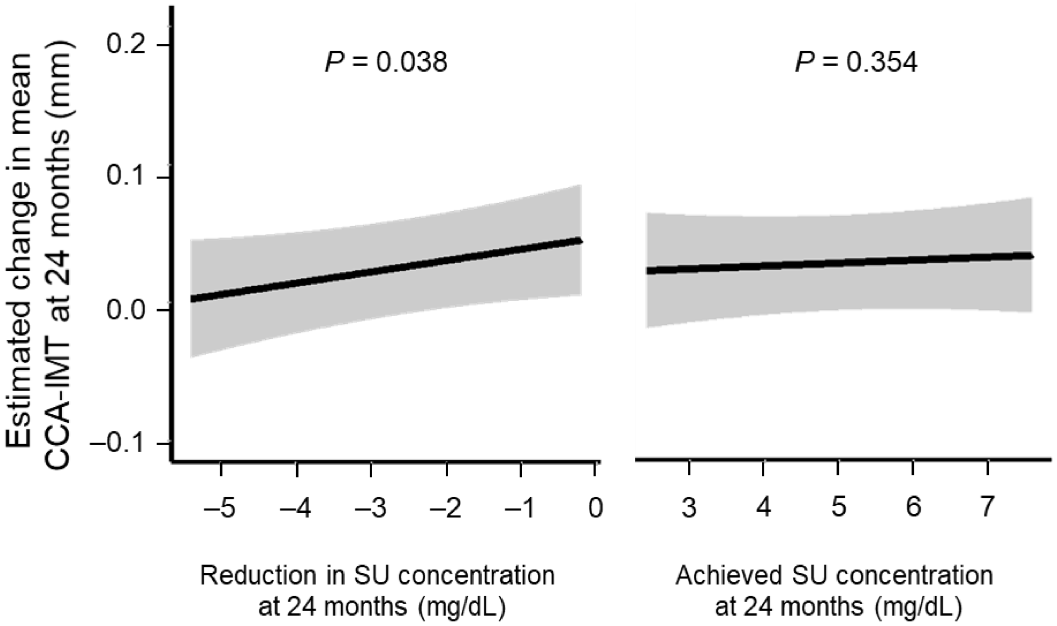

CCA-IMT, common carotid artery intima-media thickness; CVD, cardiovascular disease; SU, serum urate.

Figure S4. Association between SU levels and mean CCA-IMT values at 24 months in the subgroup without previous gouty arthritis

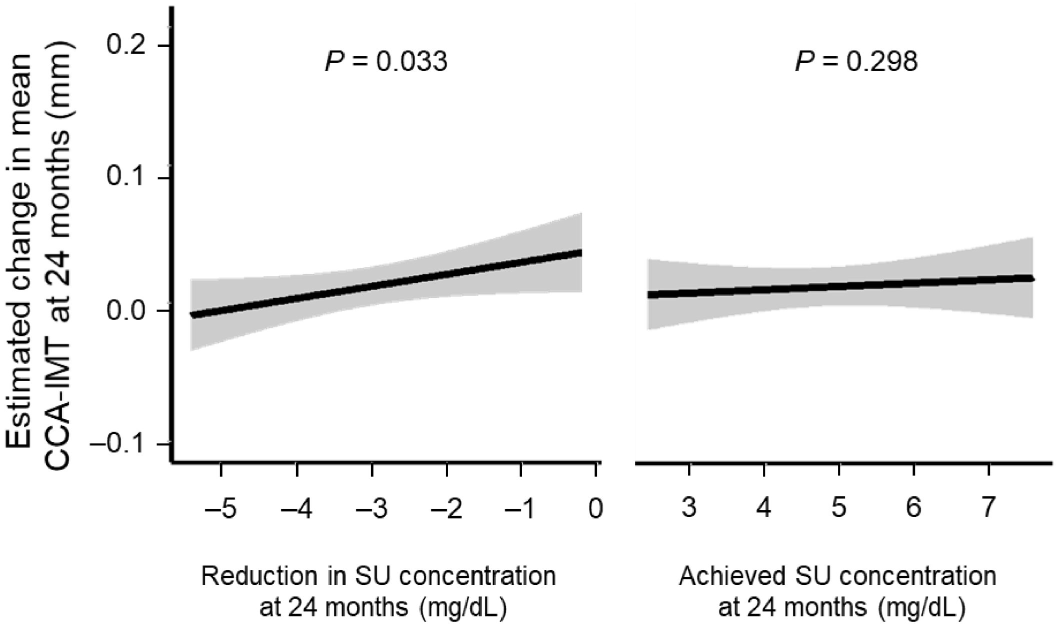

N=199

CCA-IMT, common carotid artery intima-media thickness; SU, serum urate.

Table S1. Dose of febuxostat at 24 months

Overall (n=204)	Median 20 [interquartile range 10, 40] mg daily	
**Daily dose**	**n (%)**	**n (%)**
0 mg	10 (4.9)	124 (60.8)
10 mg	65 (31.9)	
20 mg	49 (24.1)	
30 mg	4 (2.0)	80 (39.2)
40 mg	65 (31.9)	
60 mg	11 (4.9)	
